# Differential Expression Profiles and Function Prediction of Transfer RNA-Derived Fragments in High-Grade Serous Ovarian Cancer

**DOI:** 10.1155/2021/5594081

**Published:** 2021-03-30

**Authors:** Buze Chen, Sicong Liu, Haihong Wang, Guilin Li, Xiaoyuan Lu, Hao Xu

**Affiliations:** ^1^Department of Gynecology, The Affiliated Hospital of Xuzhou Medical University, Xuzhou, 221000 Jiangsu, China; ^2^Graduate School, Xuzhou Medical University, Xuzhou, Jiangsu 221000, China; ^3^Department of Gynecology, Maternal and Child Health Care Hospital Affiliated to Xuzhou Medical University, Xuzhou, 221000 Jiangsu, China; ^4^Department of Gynecology, Huangshi Love & Health Hospital Affiliated to Hubei Polytechnic University, Huangshi, 435000 Hubei, China

## Abstract

**Background:**

The present study is aimed at providing systematic insight into the composition and expression of transfer RNA (tRNA) derivative transcription in high-grade serous ovarian cancer (HGSOC).

**Methods:**

tRNA derivative expression profiles in three pairs of HGSOC and adjacent normal ovarian tissues were conducted by tRNA-derived small RNA fragment (tRF) and tRNA half (tiRNA) sequencing. The differentially expressed tRFs and tiRNAs between HGSOC and paired adjacent normal samples were screened. The targeted genes of differentially expressed tRFs and tiRNAs were screened. The Gene Ontology (GO) and the Kyoto Encyclopedia of Genes and Genomes (KEGG) of target genes of tRFs and tiRNAs were analyzed.

**Results:**

There are a total of 20 significantly upregulated and 15 significantly downregulated tRFs and tiRNAs between the cancer group and the paracarcinoma group. The upregulated tRFs and tiRNAs are mucin-type O-glycan biosynthesis, glycosphingolipid biosynthesis, the glucagon signaling pathway, the AMPK signaling pathway, maturity-onset diabetes of the young, glycosphingolipid biosynthesis, the insulin signaling pathway, insulin resistance, leukocyte transendothelial migration, starch, and sucrose metabolism. The downregulated tRFs and tiRNAs are other glycan degradation, vitamin digestion and absorption, fatty acid elongation, and biosynthesis of unsaturated fatty acids.

**Conclusions:**

There are significantly expressed tRFs and tiRNAs in HGSOC tissues, and these may provide potential diagnostic biomarkers and therapeutic targets for HGSOC.

## 1. Background

Ovarian cancer (OC) is one of the deadliest gynecological malignancies in the world [1, 2]. High-grade serous ovarian cancer (HGSOC) is the most frequent and deadly type of epithelial ovarian carcinoma, accounting for 75% of ovarian cancer cases [3]. The early stage of OC is under a 5-year survival of 93%. Unfortunately, the majority of OS patients are diagnosed at the III or IV stage, for which the 5-year survival is much lower. Therefore, looking for biomarkers and effective molecular targets for HGSOC is essential.

tRNA-derived fragments (tRFs) and tRNA halves (tiRNAs) are small noncoding RNAs derived from precursor tRNAs or mature tRNAs [4]. Transfer RNAs (tRNAs), housekeeping products, are a fundamental component of the translation machinery by acting as adapters in protein synthesis [5]. tRNA derivatives include tRNA halves (tiRNAs) and tRNA-derived small RNA fragments (tRFs). Transfer RNA-derived fragments (tRFs), 14–35 nucleotides (nt), are a novel class of noncoding RNA rooted in tRNAs [6–8]. tRFs were classified into tRF-5, tRF-3, and tRF-1 in prostate cancer [9]. tRFs play pivotal roles in cell proliferation, DNA damage response, tumor progression, and neurodegeneration via regulation of gene expression [10, 11]. Since tRFs can bind to Argonaute proteins (similar to miRNAs) and Piwi proteins (similar to piRNAs), their disruption may play a key role in cancer by regulating gene expression at different levels [12]. Recently, tRFs have been recognized as novel potential biomarkers and cancer treatment target [13].

tRNA-derived fragments are dysregulated, which could be involved in the pathogenesis and progression of ovarian endometriosis [14]. There were significantly expressed tRNA-derived fragments in breast cancer tissues, which are hopefully to become biomarkers and valuable researches in this area [15]. tRFs play crucial roles in the development of colon cancer [16]. The expression profile of tRNA-derived fragments was analyzed in pancreatic cancer [17]. Read in conjunction, these researches showed a functional role of tRNA derivatives in tumorigenesis.

In the present study, we screened tRNA derivative profiles of HGSOC patients using high-throughput tRF and tiRNA sequencing. Then, we utilized an integrative strategy to develop functional tRF and tiRNA-mRNA regulatory networks by combining the reverse expression relationships between tRFs and tiRNAs and targets and computational predictions. Our study may provide clues to gain a better understanding of the potential roles of tRNA derivatives in the pathogenesis of HGSOC.

## 2. Methods

### 2.1. Samples

Three pairs of HGSOC patients and adjacent normal ovary tissues were harvested from patients at the Department of Gynaecology and Obstetrics, Xuzhou Medical University Affiliated Hospital. The adjacent normal ovary tissue was defined as the paracarcinoma group, and the tumor tissue was defined as the cancer group. The diagnosis of HGSOC was independently affirmed by two pathologists. The patients were not given any radiotherapy or chemotherapy before surgical resection. All tissue samples were snap-frozen and kept in liquid nitrogen. This study was approved by the ethics committee of Xuzhou Medical University Affiliated Hospital, and written informed consent was issued by all patients. The information including age, years, gender, tumor size, tumor stage, and pathological description of patients was recorded.

### 2.2. RNA Extraction, Library Preparation, and tRF and tiRNA Sequencing

We followed the methods described before [18]. Total RNA was extracted and checked as the reference [19]. tRNA-derived fragments (tRFs and tiRNAs) are heavily decorated by RNA modifications that influence small RNA-seq library construction. We use the rtStar™ tRF and tiRNA Pretreatment Kit (AS-FS-005; KangChen Bio-tech, Shanghai, China) to do the following treatments before library preparation for total RNA samples: 3′-aminoacyl (charged) deacylation to 3′-OH for 3′adaptor ligation, 3′-cP (2′,3′-cyclic phosphate) removal to 3′-OH for 3′adaptor ligation, 5′-OH (hydroxyl group) phosphorylation to 5′-P for 5′-adaptor ligation, and m1A and m3C demethylation for efficient reverse transcription. Sequencing libraries are size-selected for the RNA biotypes to be sequenced using an automated gel cutter. The libraries are qualified and absolutely quantified using Agilent BioAnalyzer 2100. For standard small RNA sequencing on Illumina NextSeq instrument, the sequencing type is 50 bp single-read.

### 2.3. Identification of tRFs and tiRNAs Associated with HGSOC

Comprehensive data and result of statistical analyses are provided in the Arraystar tRF and tiRNA-seq data analysis package. The differentially expressed tRFs and tiRNAs (DETs) were screened based on the count value with R package edgeR [20]. Principal component analysis (PCA), pie plots, Venn plots, hierarchical clustering, scatter plots, and volcano plots were made in R 3.5.1 or Perl environment.

### 2.4. Target Gene Prediction of DETs

miRanda is a dynamic planning algorithm based on RNA secondary structure and free energy to discover sites of any seed type [21]. TargetScan, based on the fit of mRNA to miRNA expression profile data, finds some biologically significant site sequence signatures and a relatively conservative scoring model [22, 23]. It can only search for sites that are perfect matches for nucleotides 2-7 such as m8, 7mer-m8, and 7mer-a1. By combining the two algorithms, the advantages of both algorithms are integrated and the presentation of the results is improved.

### 2.5. GO and KEGG Analyses of Targeted Genes of DETs

The DAVID version 6.8 database (http://david.ncifcrf.gov/) is a powerful tool that helps exploiting the functions of the genes of interest. As tRFs and tiRNAs can act on target genes to downregulate their expression, DAVID was used to do the Kyoto Encyclopedia of Genes and Genomes (KEGG) and Gene Ontology (GO) analysis on the target genes. The lower the *P* value, the greater the significance (*P* ≤ 0.05 was suggested).

## 3. Results

### 3.1. Overview of tRF and tiRNA Expression Profiles in HGSOC

**Figure 1:**
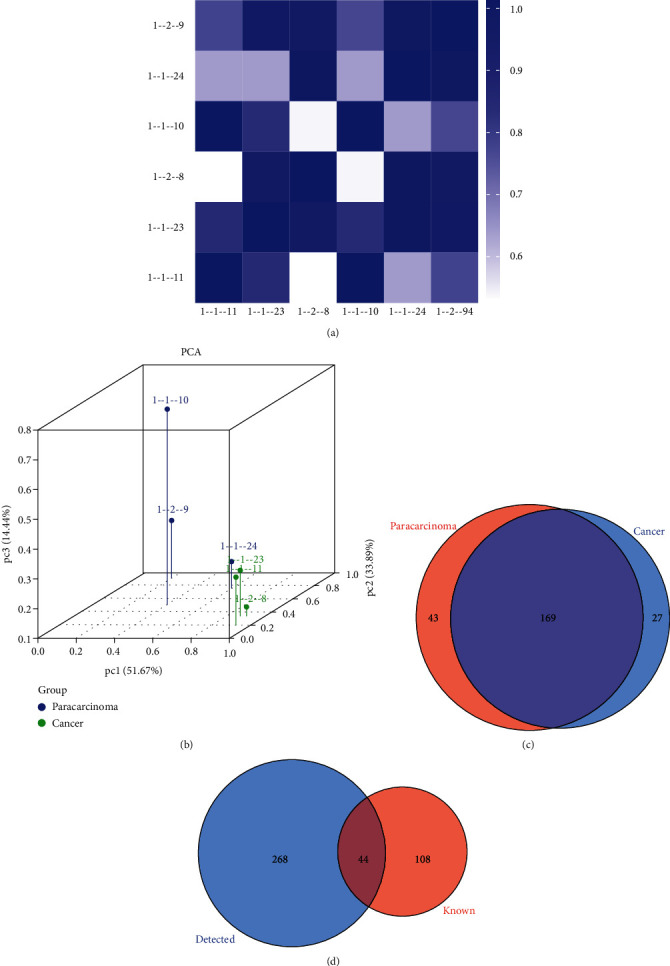
The expression level analysis. (a) Heat map of the correlation coefficient from all samples. The color of the panel is the correlation coefficient of the two samples. The blue represents the two samples with a high correlation coefficient, and the white represents the minimal similarity of the two samples. (b) PCA of strongly expressed tRFs and tiRNAs showed a complete separation of HGSOC and normal controls. PCA: principal component analysis. (c) Venn diagram founded on the number of commonly expressed and specifically expressed tRFs and tiRNAs. This diagram is the number of tRFs and tiRNAs which were expressed in both groups and indicated the number of specifically expressed tRFs and tiRNAs. (d) A total of 312 tRNA derivatives were identified by tRFs and tiRNA-seq in this study, and 268 of them were identified as novel tRNA derivatives that had not been annotated in the tRFdb database.

**Figure 2:**
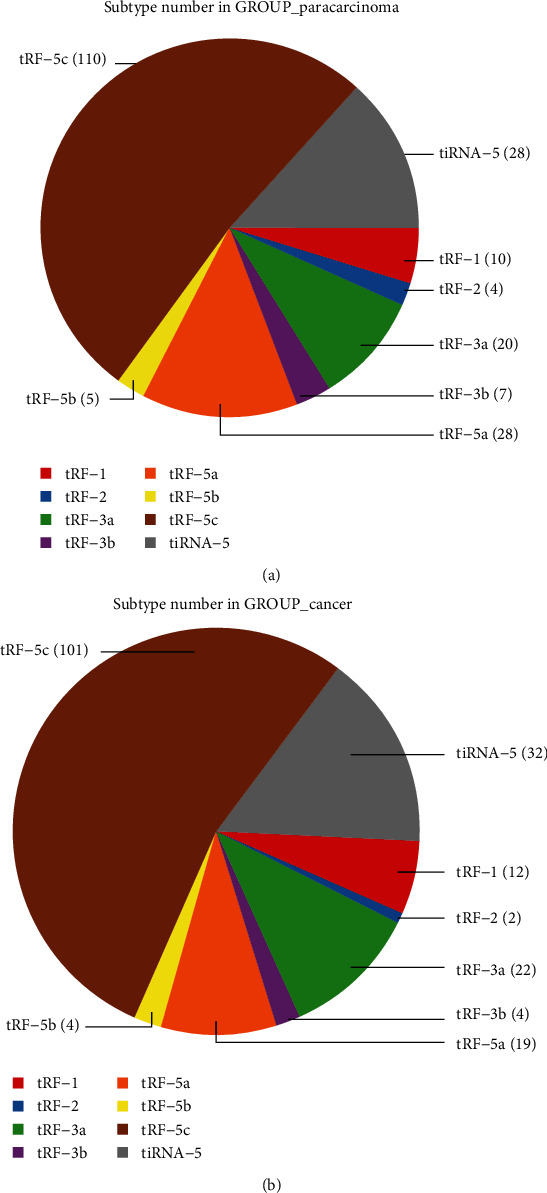
Pie charts of tRF and tiRNA subtype. (a) Pie charts of the distribution of tRF and tiRNA subtypes in the normal samples. (b) Pie chart of the distribution of tRF and tiRNA subtypes in HGSOC.

**Figure 3:**
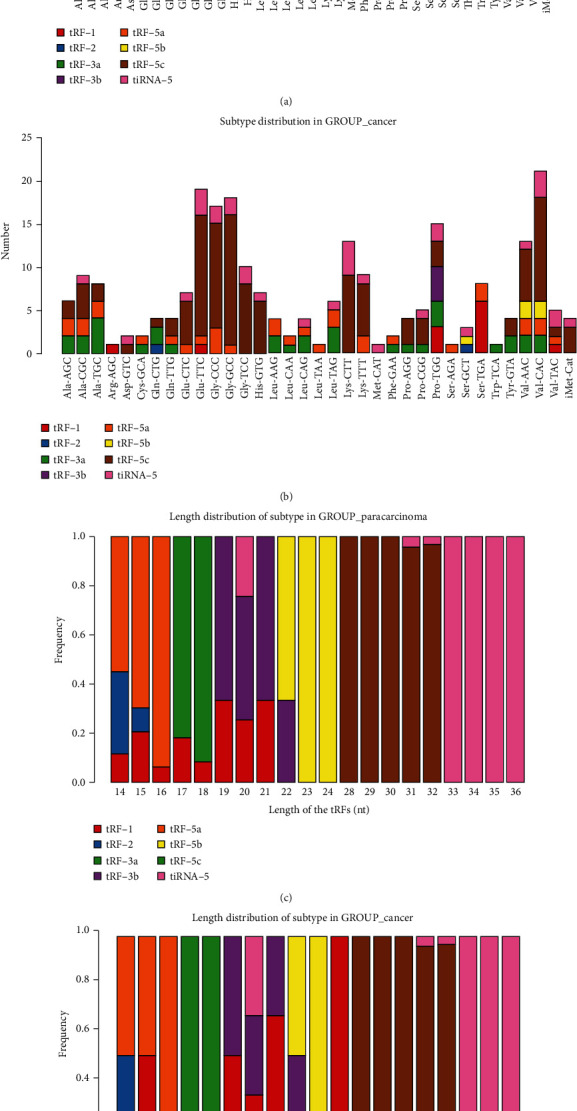
Stacked bar chart. (a) The stacked plot showed the number of tRF and tiRNA subtypes derived from the same anticodon tRNA in normal samples. (b) The stacked plot showed the number of tRF and tiRNA subtypes derived from the same anticodon tRNA in HGSOC. (c) The frequency of subtypes against the length of the tRFs and tiRNAs in normal samples. (d) The frequency of subtypes against the length of the tRFs and tiRNAs in HGSOC.

The characteristics of the three patients are listed in Table 1. We calculated the correlation coefficient between any two of the samples, which is an important evaluation criterion of the reliability and responsibility of the sample selection, showing that the two compared samples were quite similar (Figure 1(a)). In addition, PCA, a statistical method, was used for an unsupervised analysis to reduce the dimension of large datasets, and it was a useful tool to explore the sample classes based on the expression, showing a distinguishable tRF and tiRNA expression profiling among eight samples (Figure 1(b)). We identified a dysregulated expression of all 312 tRFs and tiRNAs between the paracarcinoma and cancer groups. In Figure 1(c), the Venn diagram presents a total of 169 commonly expressed tRFs and tiRNAs, 27 tRFs and tiRNAs specifically expressed in the cancer group, and 43 tRFs and tiRNAs specifically expressed in the paracarcinoma group. A total of 312 tRNA derivatives were identified by tRF and tiRNA sequencing in this study, and 268 of them were identified as new tRNA derivatives that had never been annotated in the tRFdb database [24] (Figure 1(d)). The distribution of tRF and tiRNA subtypes in HGSOC and normal colon mucosa is illustrated in Figures 2(a) and 2(b). In those tRFs and tiRNAs, the expression levels of each tRNA subtype were very different. Overall, the expression level of tiRNA-5 and tRF-1 was increased, as opposed to the paracarcinoma group. The stacked plot showed that the number of tRF and tiRNA subtypes derived from the same anticodon tRNA (Figures 3(a) and 3(b)). The frequency of subtypes against the length of the tRFs and tiRNAs is illustrated in Figures 3(c) and 3(d).

### 3.2. Identification of DETs Associated with HGSOC

**Figure 4:**
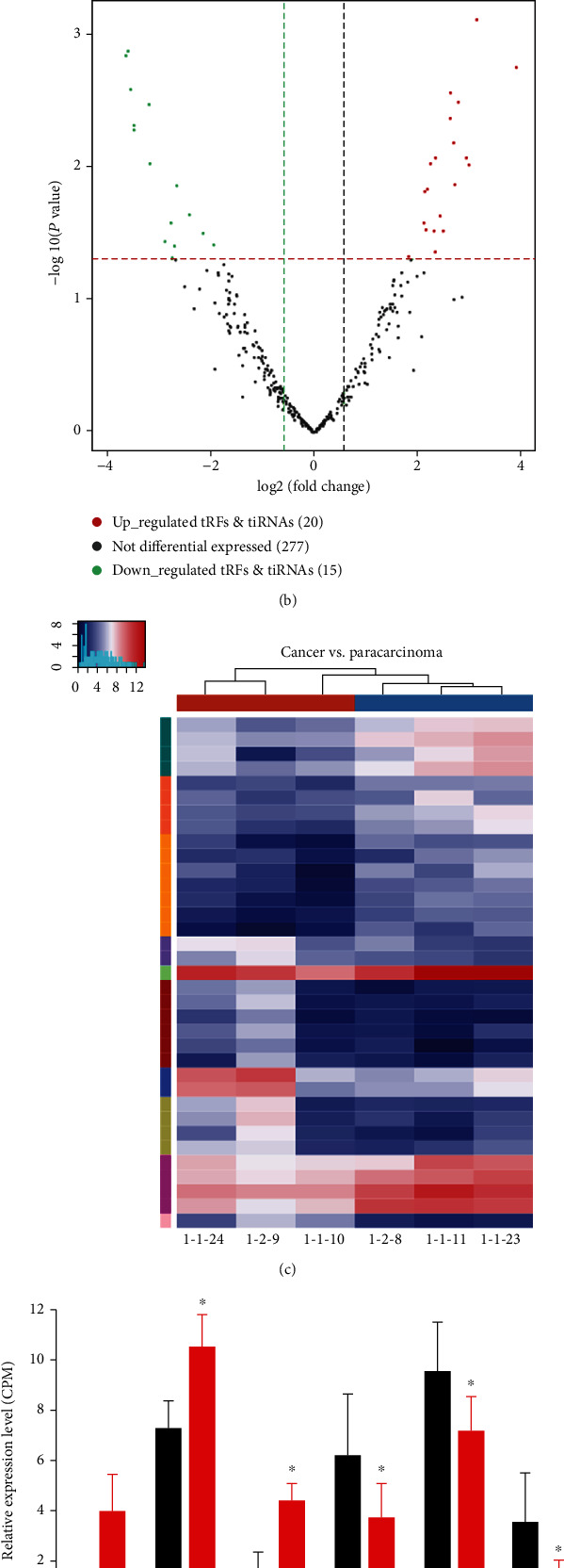
Identification of tRFs and tiRNAs related to HGSOC. (a) Scatter plot of differentially expressed tRFs and tiRNAs: dark gray points above the top line show upregulation, and light gray points below the bottom line represent downregulation; black dots indicate genes without differential expression. (b) Volcano plot of differentially expressed tRFs and tiRNAs in HGSOC. The vertical lines correspond to twofold increased and decreased expression, and the horizontal light gray line represents *P* = 0.05. The dark gray and light gray points represent tRFs and tiRNAs that were differentially upregulated and downregulated with statistical significance. (c) Heat maps of differentially expressed tRFs and tiRNAs in normal and HGSOC. (d) Relative expression levels of six most differentially expressed tRNA derivatives in HGSOC and paired normal. (e) Detailed annotation of tRF and tiRNA-mRNA interaction between tRF-Leu-AAG and its target mRNAs by computational predictions at the sequence level.

DET analyses were performed between HGSOC and coupled normal samples. There are a total of 140 upregulated and 172 downregulated tRNA derivatives (Table S1 and Figures 4(a)–4(c)). There were 20 significantly upregulated and 15 significantly downregulated tRFs and tiRNAs (Figure 4(b)) between the cancer group and the paracarcinoma group. The five upregulated and six downregulated tRNA derivatives used for follow-up studies are given in Table 2 according to fold change. These differentially expressed tRNA derivatives, including two tiRNAs and nine tRFs, were chosen to construct tRF and tiRNA-mRNA regulatory modules. Among them, tiRNA-Gln-CTG-003, tiRNA-His-GTG-001, and tRF-Ala-AGC-002 were upregulated, and tRF-Lys-TTT-012, tRF-Leu-AAG-003, and tRF-Ser-GCT-035 were downregulated in HGSOC samples reported to paired normal samples (Figure 4(d)).

### 3.3. The Targets of DETs in HGSOC

**Figure 5:**
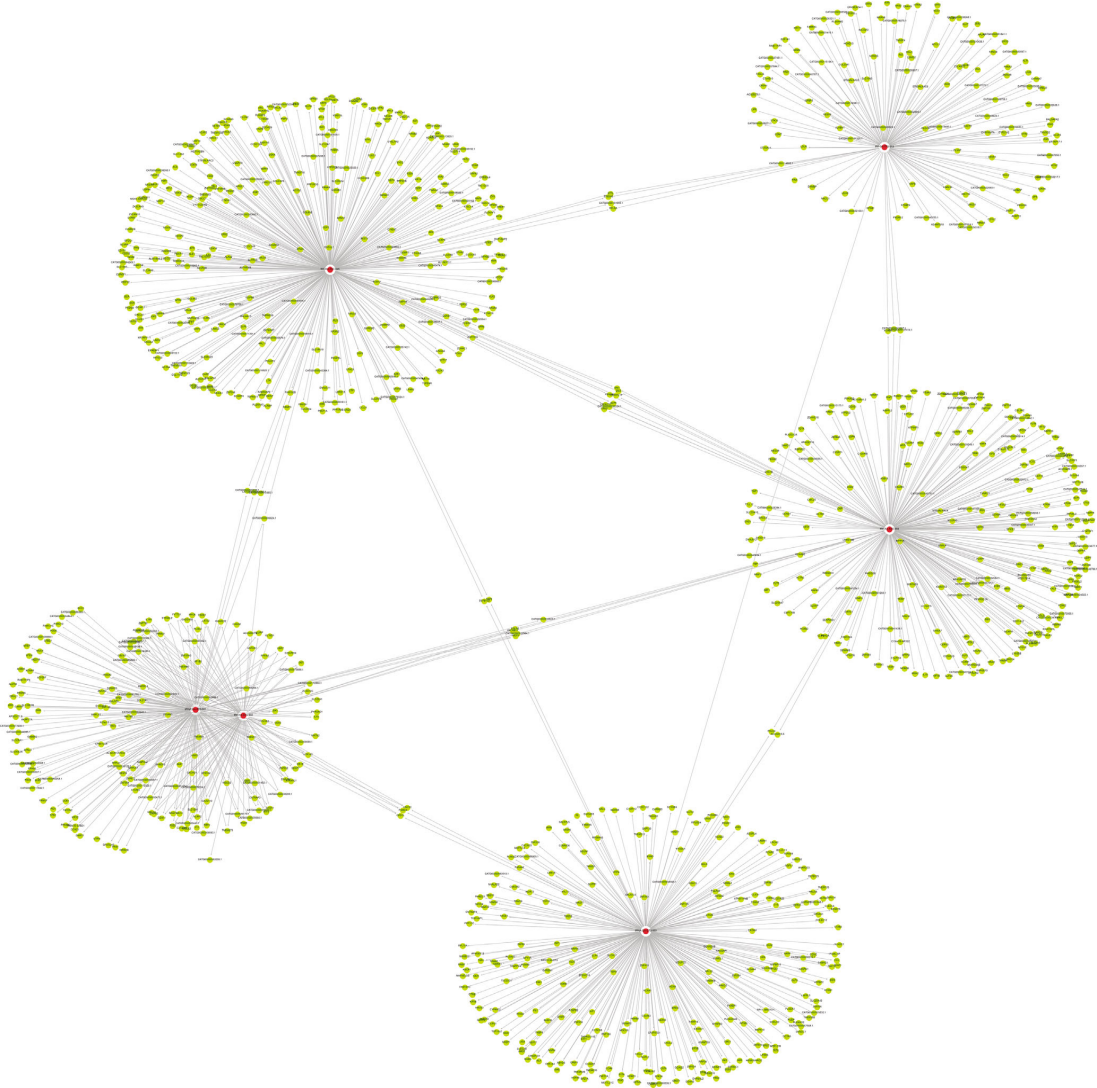
The upregulated tRF and tiRNA-mRNA network analysis. The network included the six upregulated candidate tRFs and tiRNAs and their forecast target mRNAs (nodes in red color are tRFs and tiRNAs; nodes in light-blue color are mRNAs).

**Figure 6:**
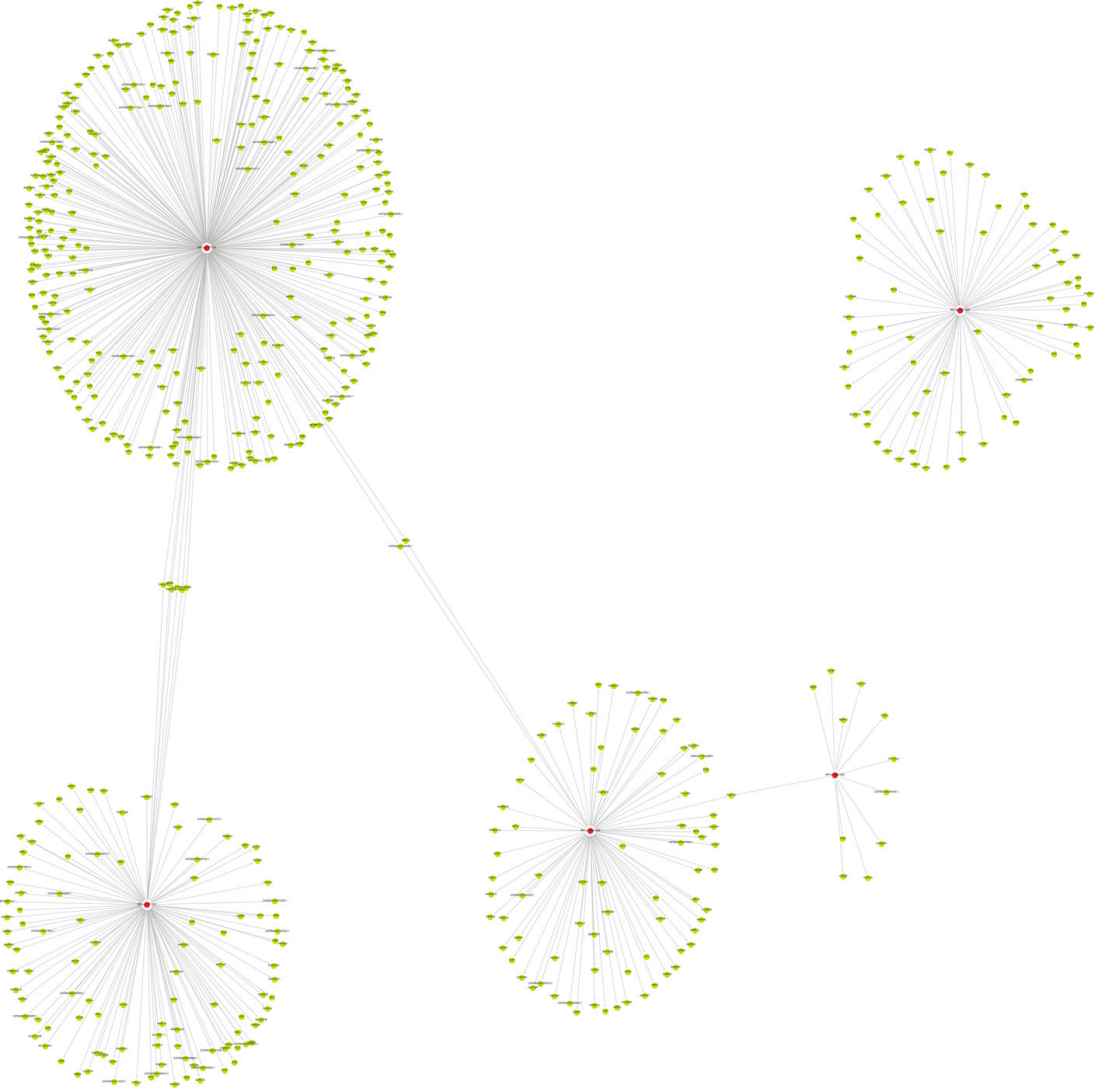
The downregulated tRF and tiRNA-mRNA network analysis. The network was the five downregulated candidate tRFs and tiRNAs and their predicted target mRNAs (nodes in red color are tRFs and tiRNA; nodes in light-blue color are mRNAs).

There are eleven significant DETs including six upregulated tRFs and tiRNAs (tiRNA-Gln-CTG-003, tiRNA-His-GTG-001, tRF-Ala-AGC-045, tRF-His-GTG-032, tRF-Ala-AGC-002, and tRF-Gln-TTG-013) and five downregulated tRFs and tiRNAs (tRF-Lys-TTT-012, tRF-Lys-TTT-014, tRF-Val-TAC-023, tRF-Leu-AAG-003, and tRF-Ser-GCT-035). tRF-Gln-CTG-003 is expected to harbor FKBP4, CACNG3, TSPAN9, APPBP2, PSMC4, AURKA, and ADGRF5 with seed sequence matching type of 8mer (Figure 4(e)). In addition, the upregulated and downregulated tRF and tiRNA-mRNA regulatory module networks were built (Figures 5 and 6).

### 3.4. GO and KEGG Analysis of DET Targets in HGSOC

**Figure 7:**
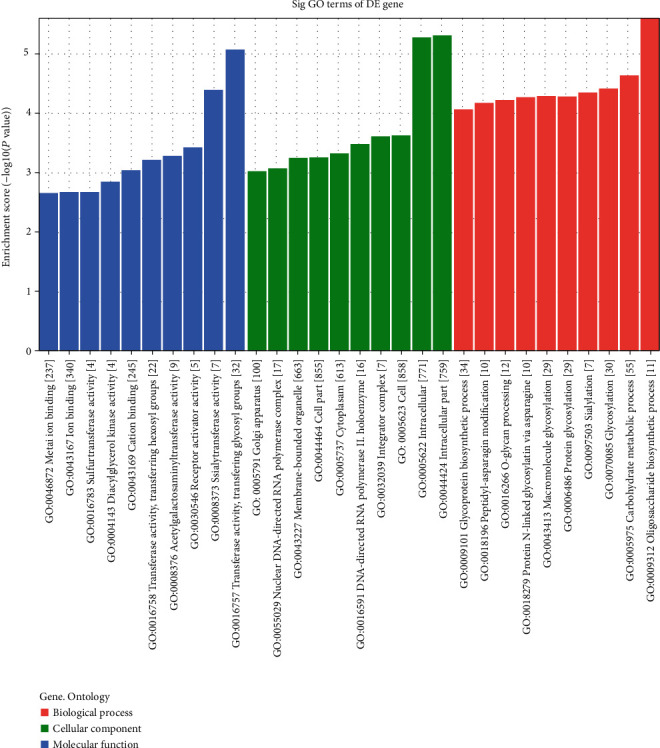
General GO annotations for CC (cellular component), MF (molecular function), and BP (biological processes) of the target mRNAs regulated by the six upregulated candidate tRFs and tiRNAs.

**Figure 8:**
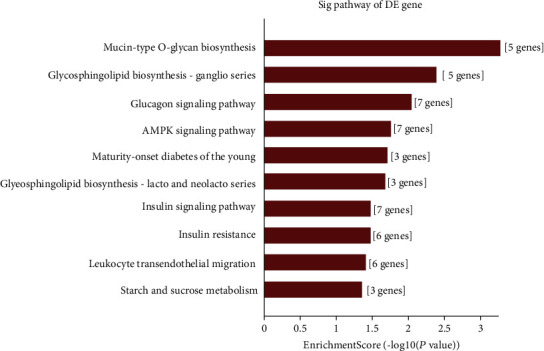
KEGG pathway analysis of the target mRNAs covered by the six upregulated candidate tRFs and tiRNAs. The bar plot shows the top 10 enrichment score values of the considerably enriched pathway.

The GO and KEGG analysis results of six upregulated tRFs and tiRNAs including tiRNA-Gln-CTG-003, tiRNA-His-GTG-001, tRF-Ala-AGC-045, tRF-His-GTG-032, tRF-Ala-AGC-002, and tRF-Gln-TTG-013, are listed in Table S2. The top ten enriched score values of the significantly enriched biological process for these upregulated tRFs and tiRNAs are oligosaccharide biosynthetic process, carbohydrate metabolic process, glycosylation, sialylation, protein glycosylation, macromolecule glycosylation, protein N-linked glycosylation via asparagine, O-glycan processing, peptidyl-asparagine modification, and glycoprotein biosynthetic process (Figure 7). The top ten enriched score values of the significantly enriched biological process for these upregulated tRFs and tiRNAs are intracellular part, intracellular, cell, integrator complex, DNA-directed RNA polymerase II, holoenzyme, cytoplasm, cell part, membrane-bounded organelle, nuclear DNA-directed RNA polymerase complex, and Golgi apparatus (Figure 7). The top ten enriched score values of the significantly enriched molecular function for these upregulated tRFs and tiRNAs are transferase activity, transferring glycosyl groups, sialyltransferase activity, receptor activator activity, acetylgalactosaminyltransferase activity, transferase activity, transferring hexosyl groups, cation binding, diacylglycerol kinase activity, sulfurtransferase activity, ion binding, and metal ion binding (Figure 7). The top ten enriched score values of the significantly enriched pathways for these upregulated tRFs and tiRNAs are mucin-type O-glycan biosynthesis, glycosphingolipid biosynthesis, glucagon signaling pathway, AMPK signaling pathway, maturity-onset diabetes of the young, glycosphingolipid biosynthesis, insulin signaling pathway, insulin resistance, leukocyte transendothelial migration, starch, and sucrose metabolism (Figure 8).

**Figure 9:**
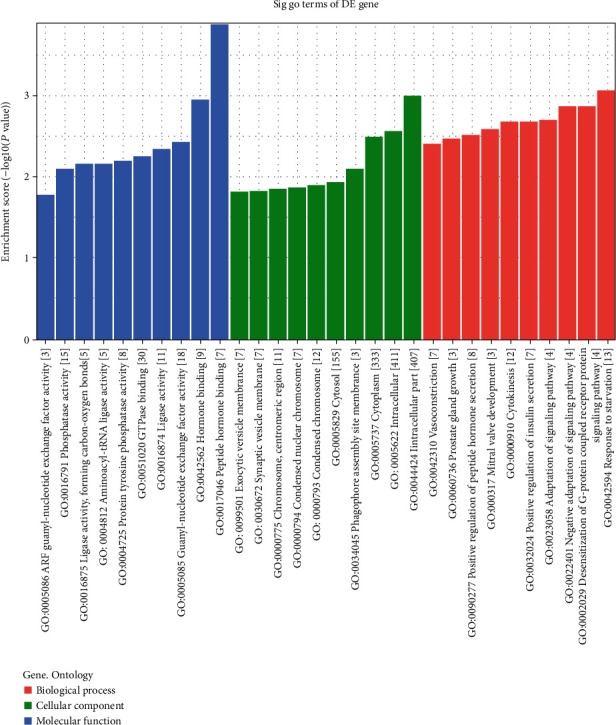
General GO annotations for CC, MF, and BP of the target mRNAs regulated by the five downregulated candidate tRFs and tiRNAs.

**Figure 10:**
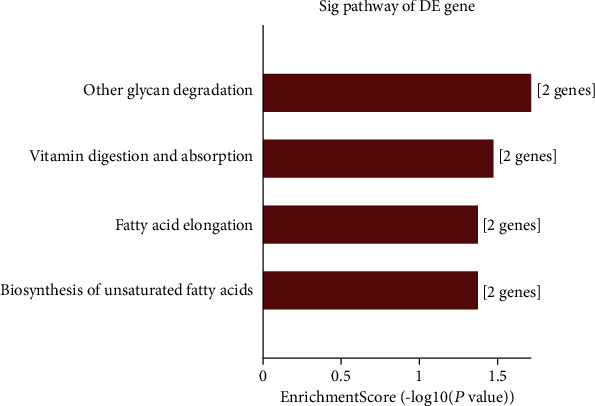
KEGG pathway analysis of the target mRNAs covered by five downregulated candidate tRFs and tiRNAs.

The GO and KEGG analysis results of five downregulated tRFs and tiRNAs including tRF-Lys-TTT-012, tRF-Lys-TTT-014, tRF-Val-TAC-023, tRF-Leu-AAG-003, and tRF-Ser-GCT-035 are listed in Table S3. The top ten enriched score values of the significantly enriched biological process for these downregulated tRFs and tiRNAs are response to starvation, desensitization of G-protein-coupled receptor protein signaling pathway, negative adaptation of signaling pathway, adaptation of signaling pathway, positive regulation of insulin secretion, cytokinesis, mitral valve development, positive regulation of peptide hormone secretion, prostate gland growth, and vasoconstriction (Figure 9). The top ten enriched score values of the significantly enriched biological process for these downregulated tRFs and tiRNAs are intracellular part, intracellular, cytoplasm, phagophore assembly site membrane, cytosol, condensed chromosome, condensed nuclear chromosome, chromosome, centromeric region, synaptic vesicle membrane, and exocytic vesicle membrane (Figure 9). The top ten enriched score values of the significantly enriched molecular function for these downregulated tRFs and tiRNAs are peptide hormone binding, hormone binding, guanyl-nucleotide exchange factor activity, ligase activity, GTPase binding, protein tyrosine phosphatase activity, aminoacyl-tRNA ligase activity, ligase activity, forming carbon-oxygen bonds, phosphatase activity, and ARF guanyl-nucleotide exchange factor activity (Figure 9). Enriched score values of the dramatically enriched pathways for these downregulated tRFs and tiRNAs are other glycan degradation, vitamin digestion and absorption, fatty acid elongation, and biosynthesis of unsaturated fatty acids (Figure 10).

## 4. Discussion

tRF and tiRNA sequencing analysis is a potent tool for the analysis of tRNA derivatives. A total of 312 tRNA derivatives, which are specific cleavage of tRNAs by specific nucleases, were marked with tRF and tiRNA sequencing in this study. In addition, we identified 268 novel tRNA derivatives from the sequencing data. These new tRNA derivatives are especially interesting for further research because of the lack of information about them in current databases [24]. Moreover, since there is still a lack of a global repository for sequences of tRNA derivatives, further annotation of these original tRNA derivatives is needed.

However, there is always a lack of systematic study investigating the involvement of tRNA derivatives in HGSOC progression by tRF and tiRNA analysis. In this study, we intended to improve the understanding of tRNA derivative expression pattern in HGSOC. In the present study, the expression level of tiRNA-5 and tRF-1 was increased, as opposed to the paracarcinoma group.

5′-tRNA-Arg-CCT, 5′-tRNA-Glu-CTC, and 5′-tRNA-Lys-TTT halves circulated at lower levels than in control subjects in patients with clear cell renal cell carcinoma, which indicated relevance as noninvasive biomarkers [25]. tRNA-Asn-ATT, tRNA-Ile-AAT, tRNA-Leu-TAA, mt-tRNA-Trp-TCA, mt-tRNA-Leu-TAA, tRNA-Pro-AGG, tRNA-Lys-CTT-1, and tRNA-Leu-AAG were associated with the clinicopathological characteristics of lung adenocarcinoma, and tRNA-Lys-CTT-1, mt-tRNA-Ser-GCT, and tRNA-Tyr-ATA were associated with cancer-specific survival [26]. In this study, the top six significantly upregulated tRFs and tiRNAs include tiRNA-Gln-CTG-003, tiRNA-His-GTG-001, tRF-Ala-AGC-045, tRF-His-GTG-032, tRF-Ala-AGC-002, and tRF-Gln-TTG-013, and the top five downregulated tRFs and tiRNAs include tRF-Lys-TTT-012, tRF-Lys-TTT-014, tRF-Val-TAC-023, tRF-Leu-AAG-003, and tRF-Ser-GCT-035. These biomolecules might offer potential diagnostic biomarkers of HGSOC for additional studies. Although PCA of strongly expressed in DMTs could completely distinguish HGSOC from normal controls, the sample size in this study was too low. Moreover, experimental validation of the occurrence of these biomolecules in the cell and tissue levels by quantitative real-time PCR is necessary for our future study.

The enriched score values of the significantly enriched pathways for DETs are mucin-type O-glycan biosynthesis, glycosphingolipid biosynthesis, glucagon signaling pathway, AMPK signaling pathway, maturity-onset diabetes of the young, glycosphingolipid biosynthesis, insulin signaling pathway, insulin resistance, leukocyte transendothelial migration, starch and sucrose metabolism, other glycan degradation, vitamin digestion and absorption, fatty acid elongation, and biosynthesis of unsaturated fatty acids. SIK2 activates the PI3K/AKT/HIF-1*α* pathway and Drp1 phosphorylation-mediated mitochondrial fission to play a critical oncogenic role in OC cells [27]. Vitamin D-binding protein regulates the insulin-like growth factor-1/Akt pathway and vitamin D receptor transcription to enhance epithelial OC progression [28]. tRF-Leu-CAG is a new diagnostic marker and potential therapeutic target in non-small-cell lung cancer, involved in regulating AURKA [29]. 5′-tiRNA-Val is a potential diagnostic biomarker for breast cancer, which is a new tumor suppressor through inhibition of the FZD3/Wnt/*β*-Catenin signaling pathway [30]. The role of differential DET-mediated AMPK signaling pathway and insulin pathway in the development of HGSOC has yet to be investigated.

## 5. Conclusion

In conclusion, our study revealed a landscape of tRNA derivative expression profiles in HGSOC. The findings may provide potential diagnostic biomarkers and therapeutic targets for HGSOC.

## Figures and Tables

**Table 1:** Patient characteristics.

Number	Cancer	Paracarcinoma	Age (years)	Gender	Tumor size (cm)	Tumor stage	Pathological description
1-YHR	1-1-11	1-1-10	66	Female	3	III	High-grade serous ovarian cancer
2-HZX	1-1-23	1-1-24	61	Female	4	III	High-grade serous ovarian cancer
3-LDM	1-2-8	1-2-9	56	Female	4	III	High-grade serous ovarian cancer

**Table 2:** Top eleven differentially expressed tRFs and tiRNAs in HGSOC.

tRF ID	tRF-seq	Type	Length	log (fold change)	*P* value	*Q* value	Regulation
tiRNA-Gln-CTG-003	GGTTCCATGGTGTAATGGTTAGCACTCTGGACTC	tiRNA-5	34	3.797045476	0.000433413	0.059015993	Up
tiRNA-His-GTG-001	GCCGTGATCGTATAGTGGTTAGTACTCTGCGTTG	tiRNA-5	34	3.36337803	0.000124177	0.039860717	Up
tRF-Ala-AGC-045	GGGGGTGTAGCTCA	tRF-5a	14	3.272871575	0.030547903	0.245146921	Up
tRF-His-GTG-032	GCCGTGATCGTATAGTGGTTAGTA	tRF-5b	24	3.128986967	0.020839879	0.215793586	Up
tRF-Ala-AGC-002	CCCCGGCACCTCCACCA	tRF-3a	17	3.051410315	0.000910286	0.059015993	Up
tRF-Gln-TTG-013	TCTCGGTGGGACCTCCA	tRF-3a	17	2.897709799	0.000827146	0.059015993	Up
tRF-Lys-TTT-012	AACACCTCTTTACAGTGACCA	tRF-3b	21	-2.578733521	0.004986343	0.114329719	Down
tRF-Lys-TTT-014	ACACCTCTTTACAGTGACCA	tRF-3b	20	-2.416218407	0.010260368	0.164678903	Down
tRF-Val-TAC-023	AACTTACACTTAGG	tRF-2	14	-2.373762725	0.008434288	0.142495079	Down
tRF-Leu-AAG-003	GGTAGCGTGGCCGAGC	tRF-5a	16	-2.353576992	0.006160394	0.12891244	Down
tRF-Ser-GCT-035	TAACAACATGGCTTTCTCACCA	tRF-3b	22	-2.338550238	0.023267934	0.219676672	Down

## Data Availability

The datasets generated and analyzed during the present study are available from the corresponding authors on reasonable request.

## Supplementary Materials

Supplementary 1 Table S1: all differentially expressed tRFs and tiRNAs in the present study.

Supplementary 2 Table S2: GO and KEGG analyses of the target mRNAs regulated by the six upregulated candidate tRFs and tiRNAs.

Supplementary 3 Table S3: GO and KEGG analyses of the target mRNAs regulated by the five downregulated candidate tRFs and tiRNAs.
